# U.S. Cattle Producer Adoption of Secure Beef Supply Plan Enhanced Biosecurity Practices and Foot-and-Mouth Disease Preparedness

**DOI:** 10.3389/fvets.2021.660857

**Published:** 2021-08-06

**Authors:** Christopher C. Pudenz, James L. Mitchell, Lee L. Schulz, Glynn T. Tonsor

**Affiliations:** ^1^Department of Economics, Iowa State University, Ames, IA, United States; ^2^Department of Agricultural Economics and Agribusiness, University of Arkansas, Fayetteville, AR, United States; ^3^Department of Agricultural Economics, Kansas State University, Manhattan, KS, United States

**Keywords:** biosecurity, cattle, disease, FMD, Secure Beef Supply

## Abstract

The prospect of a foot-and-mouth disease (FMD) outbreak in U.S. livestock populations has motivated the development of the Secure Beef Supply (SBS) Plan, which includes a comprehensive list of enhanced biosecurity practices that aim to prevent FMD transmission and facilitate continuity of business during an outbreak. While FMD poses a serious threat to livestock production in the United States, little is known about producers' uptake of the enhanced biosecurity practices included in the SBS Plan. In this study, we benchmark adoption and feasibility-of-adoption perceptions for U.S. cattle producers. Our results show adoption of the 13 enhanced biosecurity practices is generally low. Especially concerning is the low adoption of the three strongly-recommended pre-outbreak practices—having a biosecurity manager, having a written operation-specific enhanced biosecurity plan, and having a line of separation. Adoption of the pre-outbreak practices is likely low because the benefits of adopting the practices depend on a low probability, uncertain event. That said, producers who have adopted the pre-outbreak practices are more likely to have higher feasibility ratings for the remaining enhanced biosecurity practices, suggesting that adoption of the strongly recommended practices is associated with adoption of all enhanced biosecurity during an FMD outbreak. Complementarity is examined and shows that adoption of the pre-outbreak practices coincides with adoption of the outbreak-specific practices. Taken together, our results suggest that adoption of the strongly recommended pre-outbreak practices could help facilitate a quicker and more effective U.S. cattle industry response to an FMD outbreak in the United States.

## Introduction

Increased international travel and trade raises the likelihood of foreign animal disease introduction into the United States. Not everyone in the U.S. agriculture industry, however, is necessarily aware of the risks posed by foreign animal diseases. According to a National Animal Health Monitoring System cow-calf study, only 32.5% of operations claim to be fairly knowledgeable about foot-and-mouth disease (FMD) (1). As a majority of operations are not knowledgeable about FMD, it is not surprising that only 10.4% of cow-calf operations strongly agree that the United States is prepared to handle an outbreak of an animal disease not presently found in the United States (1). While actual preparedness may be better than perceived by producers, and technologies and tools continue to evolve in the preparedness and response toolkit, the lack of confidence in the United States' ability to respond to a disease like FMD is concerning.

An FMD outbreak in the United States would be nothing short of catastrophic for its livestock industries. FMD is a disease caused by a highly contagious virus that infects cattle, pigs, sheep, goats, deer, and other cloven hooved animals (2). The United States eradicated FMD within its borders in 1929; however, the virus is still present in many other countries. While not typically deadly for adult livestock, animals infected with FMD will experience diminished meat and milk production, thereby decreasing overall farm productivity and reducing revenues (2). Furthermore, an FMD outbreak would likely shut down exports of products from the livestock industry for an indefinite period of time, as U.S. access to foreign livestock and meat markets depends crucially on the disease status of domestic livestock populations (3). Taking the suspension of international trade due to an FMD outbreak into consideration, estimated cumulative losses over 10 years exceed $128 billion total for the U.S. pork and beef industries (4). An FMD outbreak would also harm other U.S. agriculture industries, with estimated cumulative 10-year losses of $1 billion for poultry producers, $44 billion for corn producers, $25 billion for soybean producers, and $2 billion for wheat producers. Critically, researchers predict significant losses, which include allied industries, irrespective of which species is initially found to have FMD, as FMD spreads among and between cattle, swine, and other cloven-hooved animals.

Upon diagnosis of FMD in the United States, state and federal officials would turn to the U.S. Department of Agriculture's (USDA) Foot and Mouth Disease Response Plan, also known as “The Red Book,” to provide guidance on responding to this very contagious livestock virus (2). The Red Book describes how slowing or stopping the spread of the virus through controlling livestock and livestock-industry movements is an integral part of responding to any instance of FMD in the United States. Specifically, a 24- to 72-h state, regional, or even national standstill notice would likely be put in place. State quarantines and hold orders (movement controls) would be established on infected premises (premises with a presumptive positive case or confirmed positive case of FMD). Control areas would be established with boundaries extending at least 10 km beyond the border of the infected premises, with strictly regulated movement into, within, and out of these areas. Exact authorities and processes for instituting movement controls in response to an FMD outbreak differ state-by-state, while in some instances the USDA may even impose a federal quarantine or other movement control by federal order (2).^1^

Should an FMD outbreak occur and animal movement be halted, restarting livestock transportation in order to maintain business continuity in the beef cattle industry would be critical to animal health and well-being, food security, and the agricultural economy. Control areas would exceed 300 km^2^ and could potentially contain many livestock operations. During an FMD outbreak, livestock movements and other necessary movements (e.g., feed movements) for affected operations would be facilitated by permits (5). Two broad categories of permits would be made available—specific permits allow movements connected with stopping the disease outbreak, while continuity of business permits pertain to continuing operations on premises within a control area that do not have FMD. Permit criteria may vary widely, but states, USDA's Animal and Plant Health Inspective Service, industry participants, and academia have exerted considerable resources to construct Secure Food Supply (SFS) Plans, which provide guidelines that may be sufficient for obtaining permits should an outbreak occur (5, 6).

SFS Plans have proven to be effective as disease outbreak response frameworks. In 2014–2015, guidelines from early versions of SFS Plans for poultry (Secure Turkey Supply Plan and Secure Broiler Supply Plan) and poultry products (Secure Egg Supply Plan) were employed to facilitate issuance of ~8,000 permits that allowed for more than 20,000 movements for premises located in control areas during the highly pathogenic avian influenza outbreak (7). The Secure Beef Supply (SBS) Plan, which is an SFS Plan specific to the beef cattle industry, helps individual cattle producers prepare to obtain permits to preserve continuity of business on their own operations should an FMD outbreak occur nearby. The SBS Plan was funded by USDA and developed by the Center for Food Security and Public Health at Iowa State University in collaboration with industry, state and federal officials, and other academic institutions with the stated goals of providing “guidance for operations with cattle that have no evidence of FMD infection” and helping those farms “prepare to meet movement permit requirements” (8).^2^

Compliance with the SBS Plan requires producers to adopt enumerated components, among which are obtaining a national premises identification number from the relevant state animal health official, preparing to monitor for FMD, and implementing (or making preparations to implement) enhanced biosecurity practices (8). A working definition of biosecurity is procedures that livestock producers can implement to prevent disease transmission across and within operations, with so-called enhanced biosecurity practices in the SBS Plan selected given known FMD exposure routes. The SBS Plan self-assessment checklist describes many enhanced biosecurity procedures, but the “Guide to the Secure Beef Supply Plan” (Guide) strongly recommends pre-outbreak implementation of having a biosecurity manager, having a written operation-specific enhanced biosecurity plan, and having a line of separation (LOS) around each operation (8). A biosecurity manager is the individual tasked with developing the operation-specific enhanced biosecurity plan. The biosecurity manager may work with a veterinarian to develop the plan, and plan templates are available online at the SBS Plan website. Finally, an LOS is a clear boundary that distinguishes off-operation movements from on-operation movements (8).

If a producer identifies a presumptive case of FMD, the Red Book specifies that enhanced biosecurity practices be employed before the positive case is even confirmed (2). The Red Book suggests that implementation of enhanced biosecurity should happen in the first 24 h after initial FMD case identification regardless of the specific details. While such a quick response would be absolutely necessary to curtail the outbreak, farmers in a control area would not have much time to react to what would certainly be a chaotic situation. However, adoption of the SBS Plan before an outbreak occurs helps farmers prepare to respond quickly (8). Notably, the three pre-outbreak practices strongly recommended by the Guide are largely preparatory. Other related, and sometimes overlapping, enhanced biosecurity practices are listed in the Guide and other operation-type specific checklists (9, 10). Adoption of these additional practices is encouraged since heightened biosecurity offers protection against endemic diseases. Additionally, preparations made before an outbreak could facilitate adoption of this enhanced biosecurity during an FMD outbreak. The SBS Plan, however, does not strictly recommend implementation of these extra practices until an outbreak occurs. For instance, in reference to cleaning and disinfection (C&D) stations, the checklist for pasture cattle suggests having “an operational, clearly marked, and equipped C&D station ready to be used in the event of an FMD outbreak” (10). The distinction is made because, depending on the practice, implementing enhanced biosecurity can be both inconvenient and expensive, and the full benefits may not be realized unless an outbreak occurs. By comparison, adoption of the three pre-outbreak practices requires relatively minimal monetary investment.

Benchmarking producer adoption of enhanced biosecurity outlined by the SBS Plan is of utmost importance to the U.S. cattle industry for many reasons, including reducing uncertainty regarding industry-wide preparedness. Identifying how many, whom, where, and why cattle producers implement biosecurity practices has value to many segments of the beef production system (and other species given the nature of FMD). Insights regarding the adoption of the three pre-outbreak practices and the relationship that has with the perceived ability to adopt other enhanced biosecurity practices are of particular importance should an FMD outbreak occur. If adoption of these pre-outbreak practices is positively correlated with perceived feasibility of adopting the other biosecurity measures during an FMD outbreak, it would suggest that the SBS Plan's recommendation of adopting the pre-outbreak practices may be effective in facilitating an FMD response that better maintains continuity of business.

## Materials and Methods

### Data

This research uses data from a 2018 survey of U.S. cattle producers. Sampling, survey administration, and data collection were done in collaboration with BEEF Magazine, a leading national publication for cow-calf operators, stocker-growers, cattle feeders, veterinarians, nutritionists and allied industries.^3^ Different survey versions were employed, with cattle operation characteristics determining which version a producer received. The three versions included surveys for a cow-calf operation, a feedlot operation, and a cattle operation. A producer qualified for the cow-calf operation survey if the operation had at least 20 beef cows in inventory, qualified for the feedlot operation survey if the operation had sold at least 50 head of fed cattle in the last 12 months, and qualified for the cattle operation survey if the operation had at least 20 head of any cattle in inventory. Cattle inventory thresholds used to determine survey eligibility were based on internal data BEEF Magazine uses for their membership subscriptions. The cow-calf operation and cattle operation versions of the survey targeted seedstock and cow-calf operations, and the feedlot operation version targeted stocker/backgrounder and feedlot operations.^4^

Printed survey invitation packets were mailed to a random sample of 1,500 producers eligible for the cow-calf survey, 1,500 producers eligible for the feedlot survey, and 2,000 producers eligible for the cattle survey. Survey invitation packets were mailed on October 22, 2018. A $1 bill, cover letter, and postage-paid return envelope were included in each invitation packet (11). Oerly, Tonsor, and Mitchell (12–14) provide additional details on survey data collection and response. Response rates were 22% for the cow-calf survey, 22% for the cattle survey, and 13% for the feedlot survey. The useable sample was reduced further, in some instances, due to limited non-response for specific survey questions. Survey questions regarding SBS Plan biosecurity adoption and operation characteristics were consistent across survey versions, enabling pooling of cow-calf and cattle operation survey respondents. We refer to them as cow-calf producers for the purposes of this analysis. The two broad categories surveyed, cow-calf producers and feedlot producers, capture most of the U.S. cattle supply chain, which is important as it allows for more complete benchmarking.

In addition to being asked questions regarding producer and operation characteristics, survey participants were presented with two lists of enhanced biosecurity practices. The first list included the SBS plan pre-outbreak practices of having a biosecurity manager (*Biosecurity Manager*) and having a written operation-specific biosecurity plan (*Biosecurity Plan*) as well as other enhanced biosecurity practices. The second list included the pre-outbreak practice of having a defined LOS (*LOS Defined*) as well as the components of an effective LOS. See Table 1 for a list of the enhanced biosecurity practices for which responses were elicited in the survey. In the survey, participants were asked to indicate whether or not they used a particular practice. Producers were also asked to provide a feasibility rating for implementation of the biosecurity practice in the event of an FMD outbreak. Feasibility ratings were presented as a Likert scale (1 = highly infeasible, 2 = infeasible, 3 = neutral, 4 = feasible, and 5 = highly feasible). The feasibility-of-adoption responses provide novel data regarding producer attitudes about adopting biosecurity measures during an FMD outbreak.

**Table 1:** Secure Beef Supply Plan enhanced biosecurity practice definitions.

**Enhanced Biosecurity Practices**	
**Biosecurity Manager**	**There is a designated biosecurity manager for the operation**
**Biosecurity Plan**	**An operation-specific, written, enhanced biosecurity plan has been developed**
Animal Origin	Animals come only from sources with documented enhanced biosecurity practices
Contingency Plan	A plan exists to manage animals in a biosecure manner on-site in the event animal movement is stopped for several weeks
Feed Storage	Feedstuffs are delivered, stored, mixed, and fed in a manner that minimizes contamination, and feed spills are cleaned up promptly
**LOS Defined**	**A line of separation is clearly defined and marked on the operation**
Access Points	Entry to the operation is restricted to a limited number of access points
Nose-to-Nose	Nose-to-nose contact with livestock on adjacent premises is prevented
Essential Individuals	Access is limited to individuals who are essential to the operation
Vehicles Clean	Vehicles, trailers, and equipment that cross the LOS are properly cleaned and disinfected
One-Way Exit	Animals leaving the operation only move in one direction across the LOS at an Access Point
Loading Area	The area designated for loading/unloading animals is not a people entry point
Areas Clean	Areas contaminated by personnel or animals after loading/unloading are properly cleaned and disinfected

*Pre-outbreak practices are in bold. Indented practices are specific components of an LOS*.

### Analysis

Mean adoption rates and mean feasibility ratings for the SBS Plan enhanced biosecurity practices are summarized and compared for both cow-calf and feedlot producers. The mean adoption rates provide a much-needed benchmark for where the industry is at in regard to biosecurity adoption aimed at known FMD exposure routes. Maintaining continuity of business during an FMD outbreak will require participation from all segments of the supply chain, thus we make comparisons across operation type for specific practices. We conduct both the benchmarking and the industry segment comparisons using cross tabulations, with results presented in Table 2.

**Table 2:** Enhanced biosecurity practice adoption proportions and mean feasibility ratings.

		**Biosecurity Manager** **(Pre-Outbreak Practice)**	**Biosecurity Plan** **(Pre-Outbreak Practice)**	**Animal Origin**	**Contingency** **Plan**	**Feed Storage**	**LOS Defined** **(Pre-Outbreak Practice)**	**Access Points**	**Nose-to-Nose**	**Essential Individuals**	**Vehicles Clean**	**One-Way Exit**	**Loading Area**	**Areas Clean**
**Cow-Calf**														
Adoption rate	Overall (*N* = 303)	0.09	0.04	0.13	0.16	0.39	0.12	0.23^*^	0.20	0.21	0.09	0.17	0.17	0.06
	1–49 cows (*N* = 36)	0.11	0.00	0.14	0.22	0.44	0.14	0.31	0.33	0.25	0.06	0.25	0.22	0.11
	50–199 cows (*N* = 152)	0.06	0.04	0.11	0.13	0.36	0.12	0.22	0.22	0.21	0.08	0.16	0.16	0.07
	200+ cows (*N* = 104)	0.13	0.06	0.14	0.18	0.42	0.12	0.21	0.13	0.21	0.10	0.14	0.13	0.04
Feasibility (*N* = 303)		2.69 (1.38)	2.84 (1.21)	3.29 (1.25)	3.30 (1.20)	3.84 (1.13)	2.90 (1.24)	3.21 (1.31)	2.85 (1.36)	3.33 (1.30)	2.98 (1.25)	3.17 (1.25)	3.07 (1.25)	2.85 (1.21)
**Feedlot**														
Adoption rate	Overall (*N* = 58)	0.14	0.07	0.17	0.24	0.47	0.12	0.34^*^		0.26	0.14	0.21	0.16	0.10
	1–999 head capacity (*N* = 47)	0.13	0.02	0.15	0.19	0.45	0.06	0.30		0.21	0.11	0.19	0.09	0.09
	1,000+ head capacity (*N* = 10)	0.20	0.30	0.30	0.40	0.50	0.30	0.50		0.50	0.30	0.30	0.40	0.20
Feasibility (*N* = 58)		2.93 (1.41)	2.88 (1.31)	3.14 (1.25)	3.31 (1.25)	3.79 (1.10)	3.07 (1.36)	3.43 (1.38)		3.34 (1.28)	3.02 (1.32)	3.16 (1.23)	2.97 (1.23)	2.88 (1.19)

**Figure 1:**
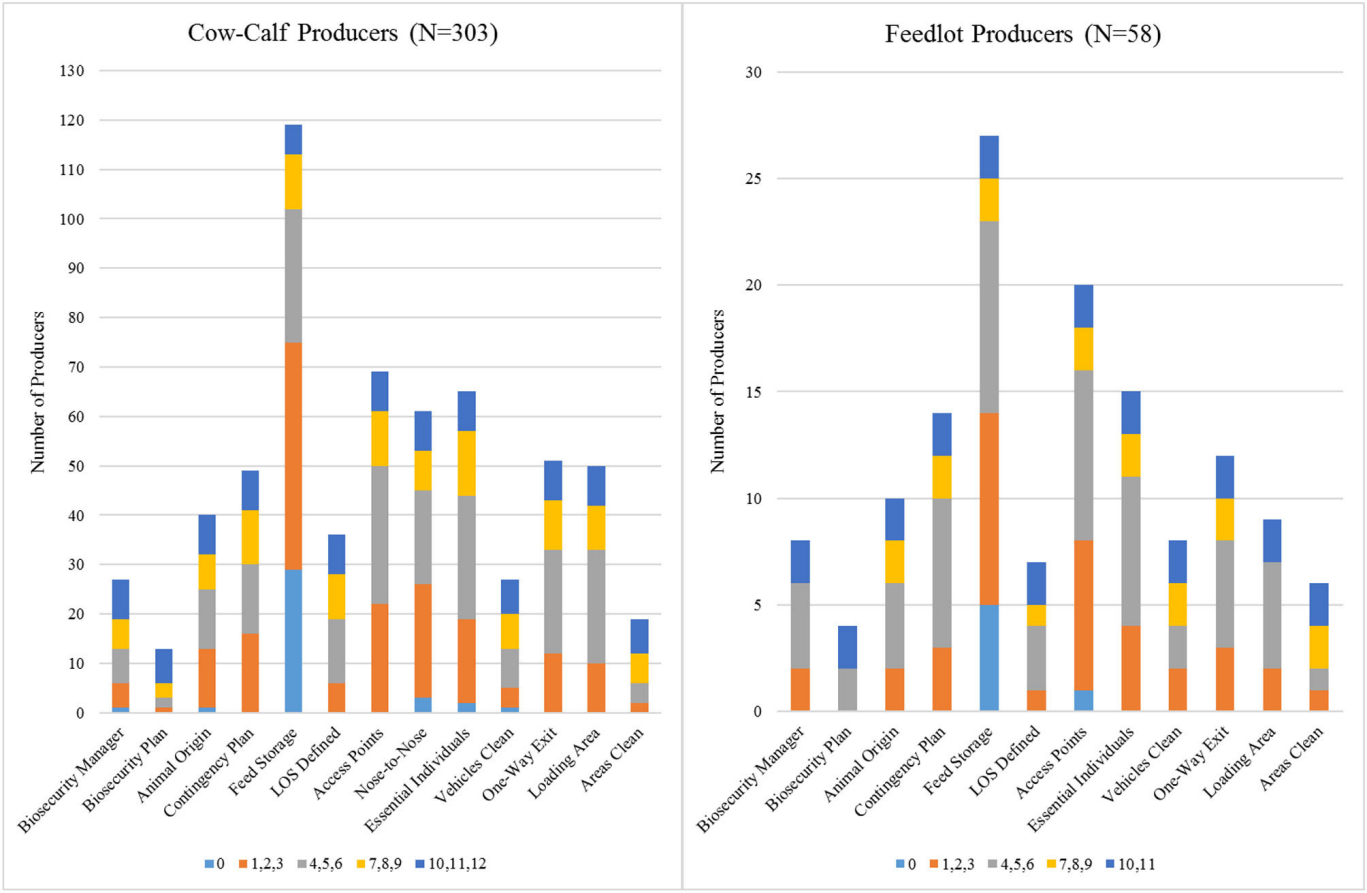
Complementarity of enhanced biosecurity practices as shown by number of other practices adopted by adoptees of a given practice. The vertical axis shows how many producers in *N* have adopted the practice named on the horizontal axis. Categories are combined for the sake of readability.

*Mean feasibility ratings are in terms of a Likert scale (1 = highly infeasible, 2 = infeasible, 3 = neutral, 4 = feasible, and 5 = highly feasible), with standard deviations in parentheses. Cow-calf and feedlot operation size categories are derived from USDA's National Animal Health Monitoring System studies (15, 16). For both cow-calf and feedlot producers, producers who did not answer the operation size question were included in the calculation of overall adoption rates and mean feasibility ratings to maintain consistency with Table 4 and Figure 1, but could not be included in the calculation of the operation-size-specific adoption rates*.

* *Represent (according to Fisher's exact-tests) statistically significant differences in overall adoption rates for cow-calf and feedlot producers at P < 0.10. No statistically significant differences in mean feasibility ratings for cow-calf and feedlot producers were demonstrated (according to Wilcoxon rank-sum tests) at P < 0.10*.

In Table 2, we also evaluate how operation size is correlated with enhanced biosecurity practice adoption for both cow-calf and feedlot producers. Benchmarking biosecurity adoption conditional on operation size is important because, in the United States, relatively few cow-calf and feedlot operations control most of the cattle inventory (17). This means that overall adoption may not provide a true understanding of industry preparedness for an FMD outbreak if, for instance, overall rates are low, but most of the largest operations have adopted the enhanced biosecurity practices. Previous literature provides some suggestive evidence as there appears to be economies of size in biosecurity adoption (18, 19).

The literature also shows correlations between geographic location and cattle producer adoption behavior and perceptions (20–22). Beef cow inventory and operations, in particular, are widely dispersed throughout the United States. These operations interact with widely diverse human, ecological, and climatic environments in their respective regions that could impact production practice choices (23). For example, SBS Plan biosecurity materials highlight that cleaning and disinfecting “can be difficult in the winter in northern climates” (24). Potential solutions such as building a sheltered cleaning and disinfecting station could be prohibitively expensive, especially if it is only employed in the event of an FMD outbreak (24). Less obvious, but equally important for preserving continuity of business during an FMD outbreak are legal environments that vary according to jurisdiction (6). For instance, according to currently published state guidance, Kansas intends to require permits for all movements state-wide following any instance of FMD in North America, which is a much more stringent permitting policy than other states (6, 25). To benchmark possible regional differences for enhanced biosecurity adoption, Table 3 presents, by region, adoption rates for the three pre-outbreak practices.

**Table 3:** U.S. beef cow inventory, operations with beef cows, and pre-outbreak biosecurity practice adoption for cow-calf producers by region.

	**Regional Totals**		**Cow-Calf Adoption (** ***N*** **= 302)**		
**Region**	**Head (1,000 s)**	**Operations (number)**	**Biosecurity Manager**	**Biosecurity Plan**	**LOS Defined**
Cornbelt (IA, IL, IN, MO, OH)	3,858	109,918	0.16	0.05	0.11
Northern Crescent (CT, DE, MA, MD, ME, MI, MN, NH, NJ, NY, PA, RI, VT, WI)	1,182	63,930	0.05	0.03	0.05
Northern Plains (KS, ND, NE, SD)	6,123	62,247	0.05	0.03	0.05
Northwest (AK, AZ, CA, CO, HI, ID, MT, NM, NV, OR, UT, WA, WY)	6,216	90,479	0.09	0.02	0.09
South (AL, AR, FL, GA, KY, LA, MS, NC, SC, TN, VA, WV)	7,269	222,142	0.12	0.06	0.27
Southern Plains (OK, TX)	6,669	180,330	0.08	0.06	0.14
Total	31,317	729,046	0.09	0.04	0.12

*Regions used in the analysis were adapted from Schulz and Tonsor (21), who use USDA Economic Research Service (ERS) farm production regions combining the lake states and northeast regions (Northern Crescent), the mountain and Pacific regions (Northwest), and the southeast region, Appalachia region, and delta states (South). Inventory (head) numbers are January 1 estimates from the USDA-NASS Cattle report (28). Operation numbers are from the 2017 Census of Agriculture (29)*.

In addition to the primary objective of benchmarking SBS Plan biosecurity adoption, the SBS Plan strongly recommending pre-outbreak adoption of certain practices suggests another specific objective for this study. Namely of interest is how adoption of the three pre-outbreak practices correlates with producers' perceived feasibility of adopting additional biosecurity practices should an FMD outbreak occur. The survey data allows for this unique analysis, which we perform using cross tabulations. Specifically, Table 4 presents mean feasibility ratings of all biosecurity practices for both adopters and non-adopters of each of the pre-outbreak practices.

**Table 4:** Enhanced biosecurity practice mean feasibility ratings conditional on adoption of pre-outbreak biosecurity practices for cow-calf and feedlot producers.

		**Biosecurity Manager** **(Pre-Outbreak Practice)**	**Biosecurity Plan** **(Pre-Outbreak Practice)**	**Animal Origin**	**Contingency Plan**	**Feed Storage**	**LOS Defined (Pre-Outbreak Practice)**	**Access Points**	**Nose-to-Nose**	**Essential Individuals**	**Vehicles Clean**	**One-Way Exit**	**Loading Area**	**Areas Clean**
**Cow-Calf (** ***N*** **= 303)**														
Biosecurity Manager	Not adopted	2.55^*^ (1.30)	2.79^*^ (1.20)	3.25^*^ (1.22)	3.26^*^ (1.18)	3.82 (1.14)	2.85^*^ (1.22)	3.16^*^ (1.31)	2.80^*^ (1.35)	3.27^*^ (1.30)	2.94^*^ (1.26)	3.13 (1.25)	3.01^*^ (1.24)	2.81^*^ (1.22)
	Adopted	4.07^*^ (1.33)	3.41^*^ (1.25)	3.74^*^ (1.40)	3.70^*^ (1.27)	4.07 (1.07)	3.44^*^ (1.34)	3.78^*^ (1.19)	3.33^*^ (1.39)	3.96^*^ (1.19)	3.41^*^ (1.12)	3.52 (1.19)	3.67^*^ (1.24)	3.26^*^ (1.10)
Biosecurity Plan	Not adopted	2.67 (1.35)	2.81^*^ (1.18)	3.28 (1.23)	3.29 (1.19)	3.83 (1.12)	2.88 (1.23)	3.18^*^ (1.31)	2.83 (1.35)	3.29^*^ (1.29)	2.96 (1.25)	3.14^*^ (1.24)	3.04^*^ (1.25)	2.82^*^ (1.21)
	Adopted	3.08 (1.89)	3.54^*^ (1.71)	3.62 (1.50)	3.62 (1.39)	3.92 (1.38)	3.38 (1.56)	3.85^*^ (1.14)	3.15 (1.46)	4.31^*^ (1.11)	3.38 (1.33)	3.77^*^ (1.17)	3.69^*^ (1.18)	3.54^*^ (1.05)
LOS Defined	Not adopted	2.65 (1.35)	2.79^*^ (1.20)	3.28 (1.24)	3.25^*^ (1.18)	3.79^*^ (1.14)	2.78^*^ (1.19)	3.08^*^ (1.30)	2.78^*^ (1.34)	3.24^*^ (1.31)	2.94 (1.27)	3.09^*^ (1.24)	2.99^*^ (1.24)	2.80^*^ (1.22)
	Adopted	2.94 (1.57)	3.25^*^ (1.27)	3.42 (1.27)	3.69^*^ (1.26)	4.17^*^ (1.00)	3.83^*^ (1.23)	4.17^*^ (0.94)	3.39^*^ (1.34)	4.03^*^ (1.00)	3.25 (1.11)	3.72^*^ (1.14)	3.72^*^ (1.19)	3.22^*^ (1.10)
**Feedlot (** ***N*** **= 58)**														
Biosecurity Manager	Not adopted	2.66^*^ (1.32)	2.74^*^ (1.29)	3.04 (1.26)	3.18^*^ (1.27)	3.68^*^ (1.11)	2.90^*^ (1.33)	3.36 (1.38)		3.24 (1.29)	2.88^*^ (1.27)	3.00^*^ (1.23)	2.82^*^ (1.21)	2.86 (1.16)
	Adopted	4.63^*^ (0.52)	3.75^*^ (1.16)	3.75 (1.04)	4.13^*^ (0.64)	4.50^*^ (0.76)	4.13^*^ (1.13)	3.88 (1.36)		4.00 (1.07)	3.88^*^ (1.36)	4.13^*^ (0.64)	3.88^*^ (0.99)	3.00 (1.41)
Biosecurity Plan	Not adopted	2.85^*^ (1.35)	2.78^*^ (1.28)	3.09 (1.25)	3.22^*^ (1.24)	3.72^*^ (1.11)	3.00 (1.36)	3.39 (1.34)		3.30 (1.27)	3.00 (1.27)	3.09 (1.23)	2.91 (1.23)	2.91 (1.15)
	Adopted	4.00^*^ (2.00)	4.25^*^ (0.96)	3.75 (1.26)	4.50^*^ (0.58)	4.75^*^ (0.50)	4.00 (1.15)	4.00 (2.00)		4.00 (1.41)	3.25 (2.06)	4.00 (0.82)	3.75 (0.96)	2.50 (1.73)
LOS Defined	Not adopted	2.75^*^ (1.37)	2.76^*^ (1.32)	3.00^*^ (1.26)	3.20^*^ (1.28)	3.71 (1.14)	2.92^*^ (1.35)	3.37 (1.37)		3.29 (1.29)	2.90^*^ (1.27)	3.06 (1.24)	2.82^*^ (1.21)	2.78^*^ (1.15)
	Adopted	4.29^*^ (0.95)	3.71^*^ (0.95)	4.14^*^ (0.38)	4.14^*^ (0.38)	4.43 (0.53)	4.14^*^ (0.90)	3.86 (1.46)		3.71 (1.25)	3.86^*^ (1.46)	3.86 (0.90)	4.00^*^ (0.82)	3.57^*^ (1.27)

*Mean feasibility ratings are in terms of a Likert scale (1 = highly infeasible, 2 = infeasible, 3 = neutral, 4 = feasible, and 5 = highly feasible), with standard deviations in parentheses*.

* *Represent (according to Wilcoxon rank-sum tests) statistically significant differences in mean feasibility ratings conditional on the adoption of the pre-outbreak practice on the vertical axis at P < 0.10*.

The analysis in Table 4 is closely related to complementarity. Simply put, complementarity with respect to biosecurity suggests that adoption of a particular practice might be made more cost effective by earlier or concurrent implementation of other biosecurity practices, or that the marginal efficacy of implementing an additional biosecurity practice may be increased by the implementation of others (18, 20, 26). To more directly examine whether or not complementarity might be a driver of increased adoption of all biosecurity, we use stacked bar charts in Figure 1 that depict the number of additional practices adopted conditional on the adoption of a given biosecurity practice. If a large number of the producers who have adopted *Biosecurity Manager*, for example, have also adopted most of the other practices, this is suggestive evidence that having a biosecurity manager is complementary with the other practices.

## Results and Discussion

### Mean Adoption Rates and Feasibility Ratings

Table 2 shows mean adoption rates and mean feasibility ratings for all SBS Plan enhanced biosecurity practices for both cow-calf and feedlot producers. For instance, 9% of cow-calf producers have adopted *Biosecurity Manager*, and the mean feasibility rating for adoption of this during an FMD outbreak is 2.69, which is somewhat infeasible if 3.0 is considered neutral. At the same time, 14% of feedlots have a biosecurity manager, and the mean feasibility rating from feedlots is closer to neither infeasible nor feasible at 2.93. Especially concerning is that so few respondents have adopted *Biosecurity Plan*, with only 4% of cow-calf producers and 7% of feedlot operators adopting this practice.

Not all adoption rates are as low as having a biosecurity plan; however, Table 2 shows that current adoption of the enhanced biosecurity practices is generally low for both cow-calf and feedlot operations−25% or lower for most of the practices. The exceptions are ensuring feedstuffs are handled properly and feed spills are cleaned up (*Feed Storage*) for both cow-calf and feedlot producers, restricting operation entry to a limited number of access points (*Access Points*) for feedlot producers, and limiting access to the operation to essential individuals (*Essential Individuals*) for feedlots. For both cow-calf and feedlot producers, *Feed Storage* has the highest adoption, which is a practice that might have higher adoption rates before an FMD outbreak for reasons other than biosecurity.

Broadly speaking, adoption rates for all biosecurity practices are similar for both cow-calf and feedlot producers, with Fisher's exact-tests showing that only *Access Points* has statistically different adoption for cow-calf and feedlot producers. Specifically, the adoption rate for *Access Points* is 23% for cow-calf producers and 34% for feedlots. Practically speaking, limiting access points is easier and less costly for feedlots given typical feedlot layouts and the smaller land area required for confined feedyards on most feedlot operations in comparison to range land or pastures for cow-calf operations (17). The lack of statistical differences in adoption could follow, at least in part, from the small sample size for feedlots as well as low adoption rates by both producer types. With this being the case, other adoption rate differences, while not statistically significant, could similarly reflect differences in day-to-day operation requirements for cow-calf and feedlot producers.

Only cow-calf operations were asked about preventing nose-to-nose contact with livestock on adjacent premises (*Nose-to-Nose*) since feedlot operations are not usually located as close to each other as cow-calf operations. The mean feasibility rating for adopting *Nose-to-Nose* during an FMD outbreak is 2.85, which is slightly infeasible and tied for third-lowest among all practices for cow-calf producers. Though implementation of this practice would be of utmost importance for a cow-calf producer in a control area should an outbreak occur on a nearby operation, the low mean feasibility rating likely reflects the difficulties of moving cattle from one pasture to another or adjusting pasture boundaries within 24 h. Implementing this practice before an outbreak could seriously impact pasture use, with low current adoption (20%) reflecting that most producers either are unaware of this biosecurity practice or consider it impractical and/or too costly until an actual disease outbreak.

Finally, mean adoption rates and feasibility ratings for the three pre-outbreak practices are generally among the lowest of all the biosecurity measures considered. This reveals that, concurrently, relatively few producers have adopted these pre-outbreak practices and they think it is relatively infeasible to do so should an outbreak occur. This makes sense—finding a biosecurity manager, while it likely requires minimal monetary investment, requires time and could be a very difficult action to execute in 24 h. Furthermore, in an outbreak scenario, many of the other enhanced biosecurity practices would be more urgent and their immediate implementation could take precedence over the pre-outbreak practices. For instance, producers in a control area would likely ensure that vehicles, trailers, and other equipment crossing the LOS are clean and disinfected (*Vehicles Clean*) before stopping to construct a written biosecurity plan. That said, having a biosecurity manager and developing a biosecurity plan may increase the feasibility of adopting *Vehicles Clean* at short notice.

### Adoption by Operation Size and Region

Table 2 also presents cow-calf and feedlot producer mean adoption rates for the enhanced biosecurity practices by operation size. Tests of statistical differences across operation size are not performed due to small sample sizes and low adoption rates, but some insights can still be gleaned. For cow-calf operations, operation size is correlated with adoption differently depending on which biosecurity practice is being considered. Consider adoption rates for *Biosecurity Plan* and *Vehicles Clean*, which are positively correlated with operation size. In comparison, adoption appears to decrease with size for *Nose-to-Nose*.

Adoption of capital intensive biosecurity practices such as *Vehicles Clean* is likely more economically viable for large commercial producers since they have more financial resources at their disposal. Furthermore, large producers could spread out the per-head costs over larger volumes of cattle (22). Adoption of managerial-intensive biosecurity practices such as *Biosecurity Plan* could also be easier for larger producers as they typically engage in less off-farm employment and work more hours on the farm (27). Conversely, practices like *Nose-to-Nose* could have lower adoption for larger producers because they could be exponentially more expensive to implement on a larger scale. It is possible that *Nose-to-Nose* could be less costly and more convenient on smaller scale cow-calf operations that require fewer and smaller pastures.

For feedlots, Table 2 shows that operations with a capacity of 1,000 or more head have higher adoption rates for every enhanced biosecurity practice compared to operations with a capacity of <1,000 head. According to the USDA, feedlots with a capacity of 1,000 head or more market more than 80% of fed cattle in the United States (17). There are, however, many more small feedlots, with 95% of U.S. feedlots having a capacity of <1,000 head (17). This makes gauging feedlot industry preparedness more difficult. Larger feedlots, while fewer in number, may be more prepared and because of this may face lesser movement restrictions, thereby helping maintain continuity of business for a large share of the U.S. cattle on feed inventory. On the other hand, smaller feedlots represent the vast majority of operations and might not be in a position to implement enhanced biosecurity and subsequently obtain necessary permits to move cattle in a timely manner. There is no obvious answer as to which measure—cattle inventory or number of operations—is a better metric for evaluating preparedness of the cattle industry. Operations and inventory can be thought of as links in a chain; a biosecurity program is only as strong as its weakest link.

Similar challenges exist as to what metric to use when benchmarking regional preparedness. Table 3 shows that adoption of pre-outbreak practices varies (sometimes widely) by region. For example, the highest adoption for *LOS Defined* is in the South, where 27% of surveyed cow-calf producers said they have adopted this practice. The lowest adoption rates for *LOS Defined* are in the Northern Crescent and Northern Plains, both at 5%. High adoption in the South is encouraging since it is the largest of the production regions in terms of cattle inventory and operations, accounting for 23% of U.S. beef cow inventory and 30% of U.S. farms with beef cows. Nearly 20% of the U.S. beef cow inventory is in the Northern Plains, compared to <4% in the Northern Crescent; however, the number of operations with beef cows in both regions is nearly equal (about 9%). If having high adoption rates in regions with more inventory is the goal, more resources should be dedicated to the Northern Plains region to help increase overall SBS Plan uptake. Alternatively, it may be desirable to dedicate more time and resources to reaching smaller producers in the Northern Crescent.

### Conditional Feasibility Ratings

Table 4 shows the relationship between current adoption of the pre-outbreak practices and perceived feasibility of adoption during an FMD outbreak. Specifically, we measure mean feasibility for all of the enhanced biosecurity practices conditional on the adoption of each of the three pre-outbreak practices. For example, cow-calf producers who have a biosecurity manager have a mean feasibility rating of 3.74 for ensuring that animals come only from sources that document enhanced biosecurity practices (*Animal Origin*). This is statistically higher than the corresponding feasibility rating of 3.25 for those cow-calf producers who do not have a biosecurity manager. This demonstrates that, in this case, having a biosecurity manager correlates with higher perceived feasibility of implementing enhanced biosecurity during an FMD outbreak.

Overall, several patterns emerge in Table 4. For nearly every practice, for both cow-calf and feedlot producers, mean feasibility ratings conditional on adoption of any of the three pre-outbreak practices are higher than the comparable mean feasibility ratings conditional on non-adoption of any of the three pre-outbreak practices. In many cases, mean feasibility ratings are statistically different. While correlation is not causation, the results suggest that adopting the three pre-outbreak practices would encourage adoption in the event of an FMD outbreak. Thus, the main result from Table 4 is that the SBS Plan strongly recommending, or even going further and incentivizing in some manner, adoption of the three pre-outbreak practices may succeed in helping producers prepare to adopt the enhanced biosecurity practices during an outbreak, as evidenced by higher perceived feasibility ratings regarding later adoption of those practices.

Some practices have feasibility ratings that are not significantly correlated with current adoption of the pre-outbreak practices. For cow-calf operations, mean feasibility ratings for *Feed Storage* are not correlated with having a biosecurity manager or biosecurity plan. Producers obtain benefits from careful feedstuff storage (e.g., reduced feed loss and spoilage) regardless of whether or not an FMD outbreak occurs (30). Storing feed properly has a cost, however. The lack of correlation between *Feed Storage* feasibility ratings and adoption of the pre-outbreak practices, in conjunction with relatively high current adoption of *Feed Storage*, suggests that for many producers the benefits must outweigh the increased storage costs irrespective of FMD considerations. Feedlot producers, who had a relatively high adoption rate for *Access Points*, demonstrate no significant correlation between feasibility ratings for that practice and adoption of any of the three pre-outbreak practices. In fact, this is the only practice for which mean feasibility is not statistically correlated with even one of the pre-outbreak practices. This result could, again, reflect the ability for feedlots to more readily limit the number of access points.

Several other findings further point to the internal consistency of the results in Table 4. First, intuitively, mean feasibility ratings for implementing a pre-outbreak practice during an outbreak are always higher among adopters of that same practice compared to non-adopters of that practice. For instance, feedlot producers who do not have a biosecurity manager have a mean feasibility rating for having a biosecurity manager during an outbreak of 2.66, which is lower than the rating of 4.63 for producers who already have a biosecurity manager. Furthermore, in both segments, producers who have a biosecurity manager think having an operation-specific biosecurity plan in an outbreak is more feasible than producers who do not have a biosecurity manager. This is important because, as discussed in SBS Plan documentation, it is the biosecurity manager who helps develop the operation-specific biosecurity plan, suggesting there is complementarity in adoption of those practices (9, 10).

### Complementarity Analysis

Results for the complementarity analysis, presented in Figure 1, extend the results from Table 4. Consider the first bar (*Biosecurity Manager*) in the cow-calf producer panel. The vertical axis shows that only 27 of the *N* = 303 cow-calf producers currently have a biosecurity manager. While those 27 producers comprise a small proportion of the sample of 303 producers, the dark blue portion of the bar shows that 8 of these 27 producers have adopted 10 or more of the other enhanced biosecurity practices. Similarly, the second bar in the cow-calf producer panel shows only 13 producers have adopted *Biosecurity Plan*, but the dark blue portion of the bar shows that 7 of these 13 producers have adopted 10 or more of the other practices. Conversely, very few cow-calf or feedlot producers have adopted the three pre-outbreak practices without adopting any other practices. Admittedly, these results are not exclusive to the pre-outbreak practices. For example, ensuring that loading areas are clean (*Areas Clean*) presents similar results. That said, there are certain practices for which complementarity does not hold. For example, 29 out of 303 total cow-calf producers and 5 out of 58 total feedlot producers adopted *Feed Storage* without adopting a single other enhanced biosecurity practice.

The results of the complementarity analysis have several potential explanations. The high rates of co-adoption among adoptees of certain practices indicates that there could be cost and/or efficiency benefits that drive adopters of the pre-outbreak practices to adopt the majority of the other practices. This explanation is not all-encompassing as fewer than 2% of cow-calf producers have adopted every enhanced biosecurity practice compared to 49% of cow-calf producers who have not adopted even a single practice (results not shown). Alternatively, it could be that many producers who adopt the pre-outbreak practices do so because it is relatively costless compared to the other 10 or more procedures they have already adopted. Either way, convincing producers to adopt the three pre-outbreak practices does not seem to reduce current adoption of other enhanced biosecurity and likely increases adoption.

### Future Outreach Efforts

Much of the outreach effort to increase SBS Plan enhanced biosecurity adoption, to-date, has been on a case-by-case, state-by-state, or regional basis. For example, in March 2020, a group of state animal health officials, beef industry representatives, and trade organizations from Colorado, Kansas, Missouri, Nebraska, Oklahoma, and Texas had a regional meeting to discuss how to best implement the SBS Plan. The first of five action items the group agreed upon was, “State-based cattle associations should become more engaged in sharing information about SBS and emergency movement permitting with producers” (6). Such emphasis on state-level outreach allows industry representatives, university extension staff, and others to leverage local information and relationships. Furthermore, focused outreach efforts could support a more effective FMD response should an outbreak occur since, as Colorado's SBS Plan highlights, “Response to an animal disease outbreak will begin at the local level” (31). That said, for all the merits of localized efforts, the benchmarking in this study shows that—at least as of 2018—SBS Plan biosecurity implementation is generally very low.

It could be the case that SBS Plan biosecurity adoption is even lower than demonstrated by this study. A limitation of survey data is the potential for selection bias. In the present study, producers who are more confident in their biosecurity practices might have been more willing to respond to surveys regarding biosecurity practices (19). This could result in higher mean SBS Plan biosecurity adoption rates and feasibility ratings in the survey samples than in producer populations. Hence, this most intuitive form of potential selection bias would augment this study's primary takeaway of low adoption of SBS Plan biosecurity. This has implications for disease control and continuity of business and suggests an even greater need to increase preparedness for FMD.

A specific result from our study that SBS Plan administrators and other proponents should consider carefully is that producers in both the cow-calf and feedlot segments of the industry are somewhat more likely to have adopted enhanced biosecurity practices that are not the three pre-outbreak practices. This could be simple economics at work. Adoption of enhanced biosecurity practices could reduce costs and/or increase revenues at all times, while producers discount the potential benefits of adopting the pre-outbreak practices because they depend on an event, i.e., an FMD outbreak occurring. The chances of an FMD outbreak occurring are small and not known with certainty, making the potential benefits of adopting the pre-outbreak biosecurity difficult to enumerate.

Further research is needed to identify the exact causal mechanisms behind producers' biosecurity adoption decisions. Detailed, farm-level data for practice-specific costs could be valuable for identifying causal economic relationships. For example, the interplay between pre-outbreak and outbreak-specific costs and benefits of making sure vehicles, trailers, and equipment that cross the LOS are properly cleaned and disinfected—and the impact this has on adoption of that practice—could be more rigorously explored given farm-level fixed and variable cost data for that practice. The authors know of no such data for the U.S. beef cattle industry, so this information would need to be collected, likely through careful producer surveys and interviews. This data collection process would also present the opportunity to illicit responses that could be leveraged in sociological and/or psychological analyses. For example, both Ellis-Iversen et al. (32) and Alarcon et al. (33) utilize interview data and socio-psychological models to identify factors driving disease control practices by livestock farmers in the United Kingdom. Studies of this kind would add to existing research and could be very important for increasing FMD preparedness, since as noted in a recent review, “human adoption and adherence to biosecurity practices is influenced by psychosocial factors and is an area of urgent research and policy consideration” (34).

Each farmer's biosecurity decisions are influenced by unique factors, economic and otherwise, including social, psychological, and contextual considerations (34). This means there is no one-size-fits-all approach to increase participation in SBS Plan biosecurity. Moving forward, however, perhaps a targeted national “train the trainer” program would be beneficial. Such a program could be used to equip regional, state, and local entities with materials that highlight the potential benefits and relatively low costs of adopting the SBS Plan's recommended pre-outbreak practices, especially in comparison to the enhanced biosecurity practices that have already been adopted. Adoption of these pre-outbreak practices could, in turn, foster producer understanding of the potential losses associated with an FMD outbreak and subsequent movement controls. The internalization of these potential costs could impact cow-calf and feedlot producers' cost-benefit calculation, thereby inducing wider adoption of all SBS Plan enhanced biosecurity practices. Such efforts, if successful in increasing SBS Plan enrollment, will not guarantee a perfect response to an FMD outbreak should one occur. However, increasing SBS Plan enhanced biosecurity is a step in the right direction for preserving continuity of business in the worst-case scenario of an FMD outbreak.

## Data Availability Statement

The data analyzed in this study is subject to the following licenses/restrictions: The datasets analyzed for this study are not publicly available because they are proprietary. Request to access these datasets should be directed to Glynn T. Tonsor, gtonsor@ksu.edu.

## Ethics Statement

The studies involving human participants were reviewed and approved by Committee on Research Involving Human Subjects/Institutional Review Board, Kansas State University. Written informed consent for participation was not required for this study in accordance with the national legislation and the institutional requirements.

## Author Contributions

CP, JM, LS, and GT made direct, substantial intellectual contribution to the work, and have given approval for its publication. All authors contributed to the article and approved the submitted version.

## Author Disclaimer

The contents are solely the responsibility of the authors and do not necessarily represent the official views of the USDA or NIFA.

## Conflict of Interest

The authors declare that the research was conducted in the absence of any commercial or financial relationships that could be construed as a potential conflict of interest.

## Publisher's Note

All claims expressed in this article are solely those of the authors and do not necessarily represent those of their affiliated organizations, or those of the publisher, the editors and the reviewers. Any product that may be evaluated in this article, or claim that may be made by its manufacturer, is not guaranteed or endorsed by the publisher.

## Footnotes

^1^An example of a federal order instituting movement controls in several counties in Texas and New Mexico following the 2002–2003 Newcastle disease outbreaks can be found at https://www.govinfo.gov/content/pkg/FR-2003-04-16/pdf/FR-2003-04-16.pdf.

^2^More information regarding the SBS Plan is available online at: https://securebeef.org/.

^3^Beef Magazine is part of the Informa Markets Division of Informa PLC. More information about BEEF Magazine is available online at: https://www.beefmagazine.com.

^4^An overview of the U.S. cattle industry, including a discussion of the cow-calf and feedlot (i.e., cattle feeding) sectors, as well as other features such as live cattle international trade, is available online at: https://www.ers.usda.gov/topics/animal-products/cattle-beef/sector-at-a-glance/.
